# Trends in detectable viral load by calendar year in the Australian HIV observational database

**DOI:** 10.1186/1758-2652-14-10

**Published:** 2011-02-23

**Authors:** Matthew G Law, Ian Woolley, David J Templeton, Norm Roth, John Chuah, Brian Mulhall, Peter Canavan, Hamish McManus, David A Cooper, Kathy Petoumenos

**Affiliations:** 1 National Centre in HIV Epidemiology and Clinical Research, University of New South Wales, Sydney, NSW, Australia; 2 Monash Medical Centre and Department of Medicine, Monash University, Clayton, VIC, Australia; 3 RPA Sexual Health, Royal Prince Alfred Hospital, Sydney, NSW, Australia; 4 Prahran Market Clinic, Prahran, VIC, Australia; 5 Gold Coast Sexual Health Clinic, Miami, QLD, Australia; 6 School of Public Health, University of Sydney, Sydney, NSW, Australia; 7 National Association of People Living With HIV/AIDS, Sydney, NSW, Australia

## Abstract

**Background:**

Recent papers have suggested that expanded combination antiretroviral treatment (cART) through lower viral load may be a strategy to reduce HIV transmission at a population level. We assessed calendar trends in detectable viral load in patients recruited to the Australian HIV Observational Database who were receiving cART.

**Methods:**

Patients were included in analyses if they had started cART (defined as three or more antiretrovirals) and had at least one viral load assessment after 1 January 1997. We analyzed detectable viral load (>400 copies/ml) in the first and second six months of each calendar year while receiving cART. Repeated measures logistic regression methods were used to account for within and between patient variability. Rates of detectable viral load were predicted allowing for patients lost to follow up.

**Results:**

Analyses were based on 2439 patients and 31,339 viral load assessments between 1 January 1997 and 31 March 2009. Observed detectable viral load in patients receiving cART declined to 5.3% in the first half of 2009. Predicted detectable viral load based on multivariate models, allowing for patient loss to follow up, also declined over time, but at higher levels, to 13.8% in 2009.

**Conclusions:**

Predicted detectable viral load in Australian HIV Observational Database patients receiving cART declined over calendar time, albeit at higher levels than observed. However, over this period, HIV diagnoses and estimated HIV incidence increased in Australia.

## Background

There has been much interest recently in the role that combination antiretroviral treatment (cART) might have in decreasing HIV transmission at a population level. A reduced HIV viral load as a consequence of cART appears to reduce the risk of heterosexual HIV transmission [1-3]. At a community level, lower rates of HIV diagnosis in San Francisco and British Columbia have accompanied lower viral loads in HIV-infected people undergoing viral load tests [4,5], and in Taiwan, rapid expansion of cART was associated with a 50% reduction in new HIV diagnoses [6].

Despite biological plausibility and the observational results, mathematical modelling studies have had inconsistent conclusions. Some studies have suggested that early HIV diagnosis and widespread cART could reduce HIV transmission at a population level [7,8], while others have suggested that relatively small changes in sexual risk behaviour could overwhelm any benefits of cART [9-11]. A key parameter in these mathematical modelling studies is the effect of cART on HIV viral load levels, with parameter estimates usually derived from cohort studies. Such parameter estimates from cohort studies are, however, often confounded with problems with missing data and patient loss to follow up.

The objective of this paper is to estimate the proportions of patients with detectable HIV viral load by calendar year in patients receiving cART in the Australian HIV Observational Database (AHOD), allowing for patient covariates and differential follow-up patterns.

## Methods

Analyses were based on patients recruited to AHOD. Detailed methods have been described previously [12], but briefly, AHOD is an observational cohort study of HIV-infected patients seen at 27 clinical sites around Australia. Data are transferred electronically to the National Centre in HIV Epidemiology and Clinical Research at the University of New South Wales, Sydney, every six months for aggregation, quality control and analysis. Core data variables include: sex; date of birth; date of most recent visit; HIV exposure; hepatitis B virus (HBV) surface antigen status; hepatitis C virus (HCV) antibody status; CD4 and CD8 counts; HIV viral load; antiretroviral treatment data; AIDS-defining illnesses; and date and cause of death.

Ethics approval was obtained from the University of New South Wales Human Research Ethics Committee and all other relevant institutional review boards, and written informed consent was obtained from all patients.

Patients were included in this analysis if they had started cART (defined as three or more antiretrovirals), and had at least one viral load assessment after 1 January 1997. Using an intention-to-treat approach, patients were considered to remain on cART if they reverted to mono or double therapy. No account was taken of changes to the antiretrovirals received. Complete treatment interruptions of more than 14 days were excluded from analyses. Any viral load tests prior to cART were also excluded. A second sensitivity analysis was limited to patient prospective follow up.

The endpoint analyzed was detectable viral load (defined as >400 copies/ml) in the first and second six months of each calendar year while receiving cART. Detectable viral load was defined as >400 copies/ml as follow up included periods when more sensitive viral load assays were not available. If a patient had multiple viral loads in a six-month period, then the viral load closest to the middle of the period was selected.

The following covariates were considered: age at baseline (<30, 30-39, 40-49, 50+ years); sex; HIV exposure (men who have sex with men, MSM + injecting drug user, IDU, heterosexual, other/unknown); AIDS prior to first cART; mono or duo antiretroviral treatment prior to first cART; HCV antibody (no/not tested, ever positive); HBV surface antigen (no/not tested, ever positive); viral load prior to first cART (0 to 365 days prior - <400, >400 copies/ml, missing); CD4 count prior to first cART (0 to 365 days prior - <100, 100-199, 200-349, 350-499, 500+ cells/mm^3^, missing); viral load in previous six-month period, including viral loads while not receiving antiretrovirals - if a viral load was missing, then the previous viral load was carried forward (<400, 400-10,000, 10,000+, missing); current CD4 count, including CD4 counts while not receiving ARVs - if a CD4 was missing, then the previous CD4 was carried forward (<100, 100-199, 200-349, 350-499, 500+); year first received cART (1993-96, 1997-99, 2000-2002, 2003+; this categorization was based on a preliminary analysis that looked at each year separately, with years of similar risk grouped together); year first HIV diagnosis (< = 1989, 1990-94, 1995-99, 2000+, not known); and time since first cART (0-9 months, 9-18 months, 18+ months).

The time since first cART covariate was not modelled in more detail beyond the early period because this would fit to patients who survive and had extended follow up. This could introduce a serious bias into the predicted rates of detectable viral load.

### Statistical methods

Repeated measures logistic regression, with generalized estimating equations methodology, was used to account for within and between patient variability. An exchangeable variance structure was assumed, but robust variances calculated, which are robust to incorrect assumed variance structure. Maximum likelihood random effects models were also fitted, and found similar covariates to be significant.

Initially, all covariates were included in the models. A backward stepwise approach was then used to reduce to a parsimonious set of statistically significant (2p < 0.05) covariates. Covariates were also excluded if there appeared to be collinearity problems (for example, associations appearing the wrong way in multivariate models).

### Predicted rates of detectable viral load

The statistical models were used to make three sets of predictions for each six-month calendar period, and predictions compared with observed rates of detectable viral load. The probability of detectable viral load was predicted for the following three scenarios:

1. All patients included in the predictions, including all patients who were lost to follow up, who had missing values, or who died. This estimates the proportions of patients with detectable viral load if they had all survived and remained on cART to the appropriate time point

2. All patients included in the predictions, but excluding patients who died from the time of death

3. Limiting predictions to patients who had a viral load test result, so predictions fitted to the analyzed data.

Scenarios 1 and 3 can be thought of as likely upper and lower limits on estimates of the proportions of detectable viral load. Scenario 1, which includes all patients who are lost to follow up, who cease cART or who die, would be an upper limit. Scenario 3, which predicts based only on the analyses data, would be a lower limit as these are patients who remain in follow up and so would generally have a better outcome. Scenario 2 was expected to lie within these two limits.

## Results

A total of 2439 patients were eligible for inclusion in the analysis. The median number of viral loads analyzed for each patient was 13 (interquartile range 7 to 19). A total of 654 patients (4.7 per 100 person years) were lost to follow up (defined as more than 12 months without a clinic visit) and 194 patients died (1.4 per 100 person years). Patient characteristics at first cART are summarized by year of first cART in Table 1. Patients who first received cART in the 1990s were more likely to have been diagnosed earlier with HIV, were slightly younger, and were slightly more likely to have been infected with HIV through male-to-male sex. Patients who first received cART in 1993-96 were much more likely to have previously received mono or duo ART than those who initiated cART in later time periods, and also initiated cART at lower CD4 counts and with more prior AIDS illnesses. Patients who initiated cART in 2000 or later were more likely to report heterosexual contact as their route of HIV infection. HCV and HBV coinfection appeared less common in patients who first received cART in 2003 or later.

**Table 1:** Patient characteristics at first cART by year of first cART

Year of first cART					
		1993-96	1997-99	2000-02	2003+
		(N = 771)	(N = 934)	(N = 280)	(N = 454)
Sex	M	735 (95%)	878 (94%)	258 (92%)	424 (93%)
	F	36 (5%)	56 (6%)	22 (8%)	30 (7%)
					
Age (years)	Mean (SD)	39 (8.9)	39 (9.9)	40 (10.0)	43 (10.2)
	Median (IQR)	37 (32,45)	37 (31,45)	39 (33,46)	42 (36,49)
					
HIV exposure	MSM	641 (83%)	728 (78%)	195 (70%)	336 (74%)
	MSM+IDU	31 (4%)	40 (4%)	16 (6%)	9 (2%)
	IDU	12 (2%)	34 (4%)	3 (1%)	8 (2%)
	Heterosexual	45 (6%)	69 (7%)	44 (16%)	65 (14%)
	Other/unknown	42 (5%)	63 (7%)	22 (8%)	36 (8%)
					
Year first HIV diagnosis	< = 1989	334 (43%)	244 (26%)	46 (16%)	36 (8%)
	1990-94	313 (41%)	290 (31%)	50 (18%)	42 (9%)
	1995-99	119 (15%)	395 (42%)	68 (39%)	67 (15%)
	2000+	0	0	109 (39%)	267 (59%)
	Not known	5 (1%)	5 (1%)	7 (3%)	42 (9%)
					
Prior AIDS	No	620 (80%)	808 (87%)	234 (84%)	407 (90%)
	Yes	151 (20%)	126 (13%)	46 (16%)	47 (10%)
					
Prior mono/Double ART	No	182 (24%)	632 (68%)	209 (75%)	377 (83%)
	Yes	589 (76%)	302 (32%)	71 (25%)	77 (17%)
					
HCV	No/not tested	673 (87%)	822 (88%)	248 (89%)	423 (93%)
	Ever positive	98 (13%)	112 (12%)	32 (11%)	31 (7%)
					
HBV	No/not tested	726 (94%)	878 (94%)	263 (94%)	444 (98%)
	Ever positive	45 (6%)	56 (6%)	17 (6%)	10 (2%)
					
Log10 viral load	Mean (SD)	4.6 (1.03)	4.5 (1.01)	4.6 (1.11)	4.4 (1.3)
	Median (IQR)	4.7 (4.0,5.4)	4.7 (3.9,5.3)	4.9 (4.2,5.4)	4.8 (3.7,5.2)
	N missing	409 (53%)	171 (18%)	44 (16%)	60 (13%)
					
CD4 Count	Mean (SD)	247 (180)	356 (241)	331 (259)	332 (238)
	Median (IQR)	220 (180,369)	330 (180,487)	283(130,486)	279 (180,429)
	N missing	187 (24%)	167 (18%)	41 (15%)	51 (11%)

The final fitted multivariate model is summarized in Table 2. Factors associated with a greater risk of detectable viral load were found to be younger age, prior mono or duo ART, a detectable previous viral load, a lower current CD4 count, and the 18-month period immediately after starting cART. First cART in more recent calendar times, and more recent reported HIV diagnosis, were found to be associated with a decreased risk of detectable viral load.

**Table 2:** Predictors of detectable viral load (>400 copies/ml) - all patients 1997-2009

		Odds ratio	95% CI	p
Age at first cART	<30 years	1.0		
	30-39	0.89	(0.77, 1.02)	0.100
	40-49	0.80	(0.68, 0.93)	0.005
	50+	0.59	(0.49, 0.71)	<0.001
				
Previous mono/double ART	No	1.0		
	Yes	1.33	(1.19, 1.48)	<0.001
				
Previous viral load	< = 400 copies/ml	1.0		
	401-10,000	9.76	(8.79, 10.85)	<0.001
	10,001+	8.65	(7.73, 9.67)	<0.001
	Missing	6.94	(5.76, 6.01)	<0.001
				
Current CD4	<100 cells/mm^3^	1.0		
	100-199	0.50	(0.42, 0.60)	<0.001
	200-349	0.33	(0.27, 0.39)	<0.001
	350-499	0.25	(0.21, 0.30)	<0.001
	500+	0.18	(0.15, 0.21)	<0.001
				
Time since first cART	>18 months	1.0		
	0-9 months	1.34	(1.19, 1.49)	<0.001
	9-18 months	1.98	(1.79, 2.19)	<0.001
				
Year of first cART	1993-96	1.0		
	1997-99	0.71	(0.63, 0.80)	<0.001
	2000-02	0.41	(0.33, 0.52)	<0.001
	2003+	0.23	(0.18, 0.29)	<0.001
				
Year first HIV diagnosis	< = 1989	1.0		
	1990-94	1.07	(0.95, 1.21)	0.247
	1995-99	0.81	(0.70, 0.93)	0.003
	2000+	0.89	(0.69, 1.14)	0.365
	Not known	0.97	(0.61, 1.56)	0.914

Covariates omitted from the model:CD4 at first cART, sex, viral load at first cART, prior AIDS, HBV, HCV, HIV exposure

**Figure 1:**
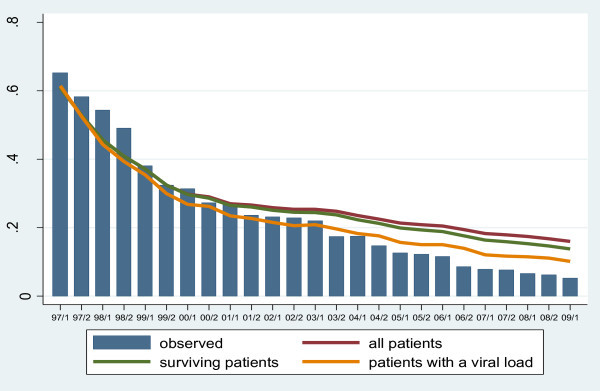
AHOD detectable viral load 1997-2009

Observed proportions of detectable viral load in patients receiving cART, by six-month calendar year periods, together with model-fitted predicted proportions, are shown for all patients combined in Figure 1. This shows a strong continuing decrease in the observed proportion of patients receiving cART with a detectable viral load, from more than 50% in 1997 and 1998 to around 7.7% in 2007, 6.3% in 2008, and 5.3% in the first half of 2009. However, the model-predicted proportions of detectable viral load are much higher. Under scenario 1, predicting for all patients including those who were lost to follow up or died, the predicted proportion in 2009 was 16.0%. The predicted proportions for scenarios 2 and 3 were 13.8% and 10.1%, respectively.

**Figure 2:**
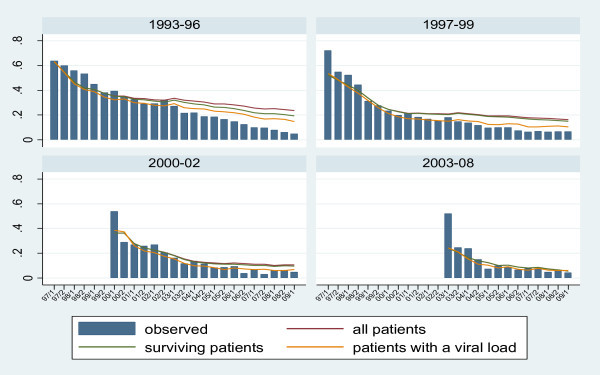
AHOD detectable viral load 1997-2009 by year of first cART

Observed and predicted proportions of detectable viral load by period of first cART are shown in Figure 2. Across all periods of first cART, there is the same strong decreasing proportion of detectable viral load down to around 5-6% in 2009. Perhaps not surprisingly, the predicted rates are much higher for patients who first received cART in earlier periods. The predicted proportions of detectable viral load under scenario 2 in 2009 were 19.4%, 14.9%, 9.8% and 5.7% for the four periods, respectively.

Sensitivity analyses were also performed based on patient prospective data only. These analyses found the same covariates to be included in multivariate models, and gave similar trends in observed and predicted proportions of detectable viral loads (data not presented).

## Discussion

The proportion of patients in AHOD with detectable viral load while receiving cART has been observed to be decreasing, to around 6% in 2009. These analyses, which adjust for patient covariates and differential follow up, suggest that the true proportions of patients in AHOD receiving cART with detectable viral load in more recent calendar time periods are higher than the simple observed proportions. The higher estimated proportion of patients with detectable viral load in adjusted analyses is mostly due to the inclusion of patients who were lost to follow up, and observed proportions should be used with caution because of this bias.

Under scenario 2 (which includes in predictions patients with unmeasured viral load or who have become lost to follow up), but censors patients who have died, the predicted proportion of patients with detectable viral load in 2009 was 13.8% compared with an observed proportion of 6.3%. Although predicted proportions of detectable viral load were higher than observed proportions, a consistent finding of our analyses was that there was no evidence of increasing proportions of patients with detectable viral load, both overall and by time of first cART. This is reassuring as it suggests that there is as yet no evidence of cohorts of HIV-infected patients running out of effective treatment options.

Our analyses specifically looked at detectable viral load by calendar time. We performed this analysis, as opposed to looking at detectable viral load from time of first cART, because of the recent interest in levels of community viral load in HIV-infected patients receiving viral load tests by calendar time, and how this might impact on HIV transmission at a population level [1-6]. In Australia, as many other countries, population-level data on rates of detectable viral load in patients receiving cART are unavailable. AHOD, a large observational cohort study that includes 15-20% of all patients in Australia receiving cART [13], is the best available source of data on this issue on which to base assumptions for mathematical models [9-11,14]. As such, analyses of this type, assessing the effect of differential follow up on observed viral load levels in AHOD, are important for developing the most accurate assumptions possible.

Combination ART is publicly funded and freely available to all HIV-infected patients in Australia. The HIV epidemic remains very largely (85%) transmitted through male homosexual sex [15], a well-educated and informed population. In uninfected homosexual men, HIV testing was reported to take place at least annually in around 60% of men in 2006, and this proportion increased between 1998 and 2006 [16]. The absolute number of HIV-infected people in Australia receiving cART has been estimated to have increased between 2000 and 2006, though the proportion of all HIV-infected people receiving cART was estimated to have increased only slightly or remained flat [17].

Finally, the analyses presented here suggest that in HIV-infected men receiving cART, HIV viral load has continued to decrease through the 2000s, albeit at slower rates than observed data suggest. This set of circumstances in Australia would appear to offer the best hope for cART to have an effect on reducing HIV transmission at a population level. However, over this period, total HIV diagnoses have increased in Australia, from a low of 718 new diagnoses in 1999 to around 1000 new diagnoses annually in 2006-2008 [15].

Mathematical models and back-projections analyses have both suggested that this reflects a real increase in HIV incidence in homosexual men [11,18]. If the decreasing trends in detectable viral load in AHOD patients receiving cART are representative of all HIV-infected patients receiving cART in Australia, then this suggests that in Australia, the likely reduction in HIV transmission risk in patients receiving cART through reduced HIV viral load is being counterbalanced by increasing infection risk due to behavioural changes. This underscores the importance of continued vigilance with existing HIV prevention strategies, including symptom awareness, early risk assessment, diagnosis and referral for care and treatment.

Mathematical modelling has been used to investigate trends in HIV incidence in Australia. Early models did suggest a decrease in HIV incidence among homosexual men during 1996 to 1998 due to the introduction of widespread cART, but that this was followed in 1998 to 2001 by a slow increase in incidence due to increasing rates of unprotected anal intercourse with casual partners while use of cART remained fairly stable [10]. More recent modelling suggested that the observed increase in HIV incidence in homosexual men in some Australian states might be explained by increasing rates of other sexually transmissible infections [11]. These models also estimated that 19% of incident HIV infections were transmitted from the estimated 3% of HIV-infected homosexual men in primary HIV infection, and that 31% of incident HIV infections were transmitted from the estimated 9% of HIV-infected homosexual men with undiagnosed infection [14].

A key limitation of our analyses is the extent to which trends in AHOD are representative of all HIV-infected people in Australia. AHOD is an observational cohort study of HIV-infected people attending clinics for their care, and recruited more patients in the late 1990s and early 2000s than in recent years. Hence trends in undetectable viral load may not reflect all HIV-infected patients receiving cART. We did stratify trends by different periods of first cART to try to assess this. AHOD represents 15-20% of HIV-infected patients receiving cART, and in terms of key epidemiological characteristics, seems reasonably representative of the wider HIV epidemic in Australia [13]. However, the estimates of trends in detectable viral load on cART in AHOD presented here are different to the true estimates of community viral load that are available in other studies [4,5], but unavailable in Australia. In particular, our analyses take no account of trends in viral load in HIV-infected people who are not receiving cART. Generalization of our results to inferences about levels of community viral load in Australia should be made with caution.

A further limitation is that AHOD, as with all observational cohorts, has missing data and some patients were lost to follow up. While we predicted trends in detectable viral load adjusted for important covariates using statistical models that allow for patients lost to follow up, there may be unmeasured and unmeasurable confounders that would affect our results. In particular, it may be that the apparent continuing decline in detectable viral load in patients receiving cART, albeit at higher levels than observed declines, is better interpreted as a plateau over the period from the mid-2000s.

## Conclusions

Our analyses suggest that in AHOD, true calendar trends in detectable viral in HIV-infected patients receiving cART are higher than observed trends when adjusted for confounding covariates and patients lost to follow up. Whether these predictions reflect true continuing decreases, or actually something more of a plateau, we feel is open to interpretation. It is reassuring that under all models, there was no suggestion of increasing detectable viral load, either observed or predicted. The fact that these decreasing trends in detectable viral load in patients receiving cART in AHOD have been accompanied by increases in HIV diagnoses and estimated HIV incidence suggests that, at least in Australia, the likely decrease in the risk of transmission from people receiving cART as a result of reduced HIV viral load is being counterbalanced by increasing risk of transmission due to behaviour changes.

## Competing interests

MGL has received research grants, consultancy and/or travel grants from: Boehringer Ingelheim; Bristol-Myers Squibb; Gilead; GlaxoSmithKline; Janssen-Cilag; Johnson & Johnson; Merck Sharp & Dohme; Pfizer; Roche; and CSL Ltd. IW has received research grants, consultancy payments, clinical support funds or honoraria from: Bristol-Myers Squibb: Gilead; Merck; and Abbott. All other authors have no competing interests to declare.

## Authors' contributions

All authors contributed to the development of the hypothesis and analysis plan. MGL and HM performed the statistical analysis. MGL wrote the first draft of the manuscript. All authors commented on drafts and approved the final version of the manuscript.
